# The Multifaceted Nature of Aminopeptidases ERAP1, ERAP2, and LNPEP: From Evolution to Disease

**DOI:** 10.3389/fimmu.2020.01576

**Published:** 2020-07-23

**Authors:** Fabiana Paladini, Maria Teresa Fiorillo, Valentina Tedeschi, Benedetta Mattorre, Rosa Sorrentino

**Affiliations:** Department of Biology and Biotechnology “Charles Darwin”, Sapienza University of Rome, Rome, Italy

**Keywords:** ERAP1, ERAP2, LNPEP, aminopeptidases, immune-mediated diseases

## Abstract

In the human genome, the aminopeptidases ERAP1, ERAP2 and LNPEP lie contiguously on chromosome 5. They share sequence homology, functions and associations with immune-mediated diseases. By analyzing their multifaceted activities as well as their expression in the zoological scale, we suggest here that the progenitor of the three aminopeptidases might be LNPEP from which the other two aminopeptidases could have derived by gene duplications. We also propose that their functions are partially redundant. More precisely, the evolutionary story of the three aminopeptidases might have been dictated by their role in regulating the renin–angiotensin system, which requires their controlled and coordinated expression. This hypothesis is supported by the many species that lack one or the other gene as well as by the lack of ERAP2 in rodents and a null expression in 25% of humans. Finally, we speculate that their role in antigen presentation has been acquired later on during evolution. They have therefore been diversified between those residing in the ER, ERAP1 and ERAP2, whose role is to refine the MHC-I peptidomes, and LNPEP, mostly present in the endosomal vesicles where it can contribute to antigen cross-presentation or move to the cell membrane as receptor for angiotensin IV. Their association with autoinflammatory/autoimmune diseases can therefore be two-fold: as “contributors” to the shaping of the immune-peptidomes as well as to the regulation of the vascular response.

In the human genome ERAP1 (Endoplasmic Reticulum Aminopeptidase 1), ERAP2 (Endoplasmic Reticulum Aminopeptidase 2) and LNPEP (Leucyl and Cystinyl Aminopeptidase) lie contiguously in 200 kilobase segment on the chromosome 5q21 and share sequence and functions as aminopeptidases. They are members of the oxytocinase subfamily of M1 Zn-metallopeptidases (1, 2). Noteworthy, their protein sequences are closely related, with LNPEP showing 43 and 49% identity to ERAP1 and ERAP2, respectively, while the two ERAP enzymes are 49% identical. These observations suggest recent gene duplication events and subsequent divergence.

ERAP1 and ERAP2 are expressed in various human tissues and are regulated by interferon-gamma (IFN-γ) (3); they reside in the endoplasmic reticulum (ER) where they trim the N-terminal peptide residues to the correct length to bind the HLA-class I molecules. In fact, cytosolic peptides are delivered through channels formed by TAP (Transporter associated with antigen processing) into the ER where they will be trimmed at their N-terminal end by the ERAPs. ERAPs have different specificities and they complement each other in shaping the antigenic repertoire: ERAP1 preferentially cleaves N-terminal hydrophobic residues whereas ERAP2 prefers positively charged amino acids. Both however cannot trim peptide bonds involving Pro. Indeed, this amino acid is frequently found at P2 in epitopes presented by HLA-I molecules sharing a Pro-permissive B pocket (4). Trimming of longer peptides probably requires the concerted action of both ERAPs and indeed ERAP1 and ERAP2 have been found to co-localize *in vivo* and to form heterodimeric complexes (5). The homologous gene LNPEP encodes a cytosolic and endosomal aminopeptidase (IRAP, Insulin-Regulated membrane Aminopeptidase) cleaving before cysteine and leucine as well as other amino acids. Endogenous peptides including arginine-vasopressin and oxytocin have been show to be processed by LNPEP. Alternative splicing results in multiple transcript variants encoding different isoforms. Interestingly, LNPEP can be found secreted in soluble form in maternal serum during normal pregnancy (6). Thanks to an additional N-terminal cytoplasmic domain, LNPEP is retained in the endosomal vesicles from where it can traffick to the cell membrane forming a type II integral membrane glycoprotein. LNPEP co-segregates with GLUT4 transporter in storage vesicles that traffic to and from the plasma membrane in insulin-responsive cells (7). Besides, LNPEP in the membrane catalyzes the final step of the angiotensinogen to angiotensin IV (AngIV) conversion and it is acknowledged as AT4 receptor (8) being in humans predominally expressed in brain and, to different extents, in heart, kidney, adrenals and blood vessels. LNPEP has a positive influence on a number of physiological and behavioral functions including blood flow, neuroprotection, synaptogenesis, long-term potentiation and memory consolidation and retrieval (9–11). LNPEP is also an essential component in the renin–angiotensin system (RAS): it degrades peptide hormones, such as oxytocin, vasopressin and angiotensin III (AngIII), and plays a role in maintaining homeostasis during pregnancy. In the evolution of human race the RAS played an important role due to its ability to control salt intake and stimulate thirst. Recent studies have unraveled roles for RAS and its main effector molecule angiotensin II (AngII) in inflammation, autoimmunity and aging (12). AngII levels can be regulated by angiotensin converting enzyme 2 (ACE2), that leads to the production of the vasodilatory heptapeptide Ang 1–7, and by other aminopeptidases including ERAPs generating AngIII (Ang 2–8) and AngIV (Ang 3–8). AngIII has similar effects to AngII, although with lower potency, in enhancing blood pressure and vasopressin release and stimulating the expression of pro-inflammatory mediators (13). AngIV exerts a protective role by increasing blood flow in the kidney and brain (12). In fact, one the most recent advance in the field, it has been the discovery of local RAS in heart, brain, pancreas, lymphatic and adipose tissue. The local RAS can operate independently or in close interaction with circulating RAS. In addition, a functional intracellular RAS has been identified highlighting several prominent effects of AngII, including pro-inflammatory, proliferative and pro-fibrotic activities (8, 14). In this context, LNPEP is involved in the inflammatory response by activating the NF-kB pathway via Ang IV (15, 16). Most interestingly, it has been demonstrated that its aminopeptidase activity is responsible for the trimming of cross-presented peptides in the endosomes of dendritic cell (DC). This observation makes LNPEP as fully belonging to the antigen presenting machinery with a prevalent role in refining extracellular epitopes. However, at the current state a contribution to the processing of intracellular epitopes traveling across the vesicles network cannot be excluded (17, 18).

## Disease Associations

The three aminopeptidases have been found associated with distinct as well as overlapping pathologies (19). This has been discussed in several reviews (2, 4, 19–21). In short, ERAP1 is associated with birdshot chorioretinopathy (22), ankylosing spondylitis (23–26) psoriasis and Behçet's disease (27) in individuals carrying the corresponding HLA-I susceptibility alleles. Interestingly, while ERAP1 haplotypes determining a higher expression associate with the first three diseases, Behçet's risk alleles induce a lower ERAP1 expression (28). Since the peptides bound to the susceptible HLA-B51 molecules carry a Pro2 or Ala2 as N-terminal anchor, it is possible that ERAP1 influences the balance of the resultant peptidomes (4, 29). In general, genetic and functional data show that the haplotypes carrying polymorphisms associated with a higher ERAP1 and/or ERAP2 expression are, in most cases, detrimental. In the case of ERAP1 this can be due to its activity as “ruler” either by destroying “protective” epitopes or by refining the susceptible peptidome (30, 31). To this regard, a recent observation has shown that in a melanoma cell, ERAP1 inhibition enhanced the predicted MHC-I binding affinity, reduced the presentation of sub-optimal long peptides and increased the presentation of many high-affinity 9–12 mers, suggesting that the baseline ERAP1 activity is destructive for many potential epitopes (32). A higher expression of ERAP2 is associated with birdshot chorioretinopathy, ankylosing spondylitis and psoriasis (4, 19, 33, 34). However, and most interestingly, this association is not in epistasis with the HLA-I susceptibility allele (35). This suggests that the two molecules do not necessarily converge in the same pathway in conferring disease susceptibility. More precisely, it allows to speculate that, while ERAP1 is fundamental in shaping the peptide repertoire for the relevant HLA-I molecules, ERAP2 can contribute different facets to disease pathogenesis. This is also suggested by the association of ERAP2 with a wider array of diseases, such as preeclampsia or IBD (36–38). At present, the relationship between ERAP1 and ERAP2 is not completely clear: there are suggestions that they can cooperate, but also that ERAP2 can regulate ERAP1 expression and activity (39, 40), modifying the peptidome and, consequently, the expression of the HLA molecules in a subtype-dependent manner (23). LNPEP has been involved in diabetes (41) and associated with psoriasis (42, 43), sharing the last feature with the other two aminopeptidases. Although not demonstrated in the literature, it is however still possible that LNPEP can contribute to disease pathogenesis by modulating the peptidome cross-presented by the HLA-I molecules (44). More interesting, although speculative, is the hypothesis that the LNPEP specific role in the RAS can have a strong impact in psoriasis. Several considerations support this hypothesis: angiotensin-converting enzyme gene, which is also a key component in the renin–angiotensin system, has been reported to be associated with psoriasis (45–47). Moreover, it has been shown that LNPEP genetic variants are associated with biological effects on vasopressin clearance and serum sodium regulation (48). Furthermore, the activation of the NF-kB cascade via Ang IV can play an additional role (15, 16). In the lack of conclusive data, we can assume that LNPEP can contribute to different aspects of the disease.

## Are LNPEP and ERAP2 Partially Interchangeable?

Here we want to consider the hypothesis that ERAP2 can exert functions affecting or at least partially overlapping with those of LNPEP, in particular in local RAS resulting from inflammation. Indeed ERAP2, besides its function as peptide trimmer, has also been found to play a role in the AngII conversion in AngIII and AngIV and consequently, in the predisposition to preeclampsia via the RAS (49, 50). It must be reminded that the association of ERAP2 with the immune-mediated diseases is in quantitative terms. Indeed, ERAP2 displays a balanced polymorphism at the SNP rs2248374 (A/G) that controls protein expression. In particular, the presence of a guanosine causes the elongation of exon 10 inducing a nonsense-mediated RNA decay, a quality-control process that destroys incorrect mRNAs. Consequently, G/G homozygotes accounting for about 25% of the population, do not express ERAP2. This has suggested that a selective pressure by infectious agents has helped to maintain a balanced selection (51). However, another possibility is that there is a redundancy in the function of the three aminopeptidases inside and outside the cells being all of them secreted. Given the function of ERAP2 in RAS, it can be hypothesized for this protein an ancillary role which makes it dispensable and even dangerous if over-expressed. This is suggested not only by the lack of ERAP2 in 25% of humans but also by its absence in rodents.

## Evolving Aminopeptidases

**Figure 1:**
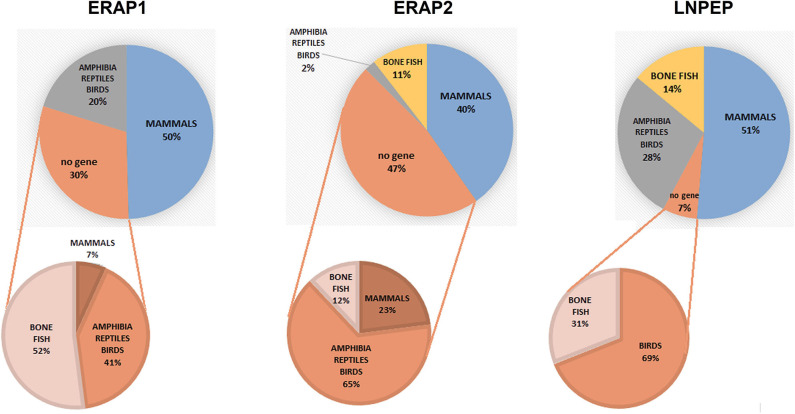
Pie charts summarizing the expression of the orthologous genes ERAP1, ERAP2 and LNPEP along the animal classes as reported by the NCBI's Gene resource (https://www.ncbi.nlm.nih.gov/search/all/?term=orthologs). Amphibians, reptiles, and birds have been merged due to the small number of available species analyzed. The composition of the negative fraction for each gene is shown in the pie charts at the bottom.

**Figure 2:**
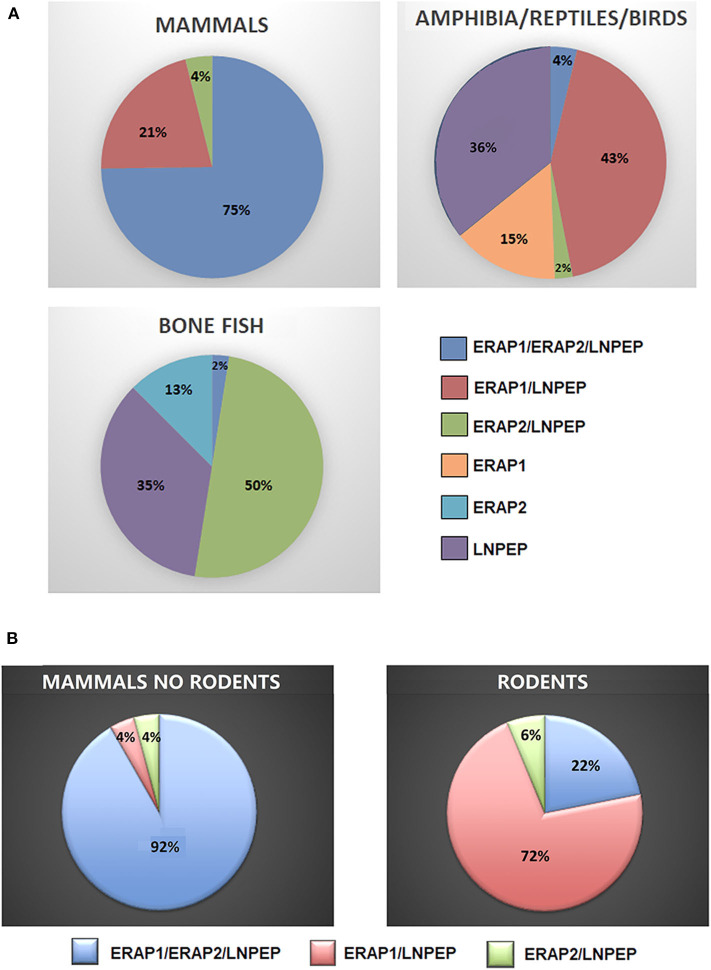
**(A)** Percentages of mammals, amphibians, reptiles, birds, and bone fish expressing ERAP1, ERAP2, and/or LNPEP genes. **(B)** Percentage of ERAP1, ERAP2, and/or LNPEP genes in the mammalian class (left) extrapolating rodents (right).

To have a hint on this intriguing question, we interrogated data banks asking what the expression of ERAP1, ERAP2 and LNPEP across the species is (248 species: 127 mammals, 61 birds, 9 lizards, 4 turtles, 4 alligators, 4 amphibia, 40 bone fish) (Supplementary Table 1). Ortholog ERAP1, ERAP2 and LNPEP genes appear in 173 (69.8%), 131 (52.8%), and 232 (93.5%) species, respectively. ERAP1 is substantially missing in bone fish (39 species) and a large part of birds (29 species). Instead, most mammals that do not express ERAP2 are rodents, which make-up to 85% of the ERAP2 negative fraction in mammals (23 rodents together with domestic cat, rabbit, koala and common wombat). As for LNPEP, however, the species that do not express this gene are predominantly birds (69%, 11 species) and bone fish (Figure 1). Overall, most of the mammals express the three genes (Figure 2A) and they usually are in the same region of the chromosome. An exception is the lack of ERAP2 in rodents (Figure 2B). It has been suggested that ERAP2 derives from a gene duplication event of ERAP1 in mammals. This appears most unlikely since ERAP2 is present in amphibia, in fishes and even in reptiles (Figure 1). Interestingly, within the rodents, *Mus pahari* (shrew mouse) carries ERAP1 in a different chromosome (chr 11) than LNPEP (chr 21). Most intriguingly, a truncated form of ERAP2 is present in this species, contiguously to LNPEP. This observation could suggest that indeed ERAP2 stems from a duplication of LNPEP and that it has been lost in rodents. Besides, mouse ERAP1 is encoded by a sequence near a putative breakpoint on chromosome 13 and LNPEP is encoded on chromosome 17. Mouse ERAP2 may therefore have been destroyed by a recombinant event. In any case, ERAP2 is apparently not required for an efficient trimming in this species. Why that happened, it remains unknown. However, it confirms that ERAP2 in the antigen processing and presentation (APP) pathway is dispensable and, most probably, redundant. ERAP1 instead is present in almost the totality of mammals whereas absent in the majority of bone fish, which express ERAP2 together with LNPEP. The birds are the most interesting case. They almost completely lack ERAP2 and the great majority (50 species) expresses LNPEP. However, there is a subset that expresses ERAP1 only, and a tiny group expressing ERAP2 only, suggesting again that there is a high degree of redundancy among these genes. ERAP2 is instead lacking in two of the three amphibia species analyzed, the only positive being *Rhinatrema bivittatum*. In reptiles LNPEP is expressed across all species whereas the expression of ERAP1 and ERAP2 appears again as alternate. In bone fish the majority of species lacks ERAP1, or both ERAP1 and ERAP2. There is however a significant percentage that expresses ERAP2 only and a very tiny subset expressing the three genes. This analysis points out a high degree of redundancy among the three aminopeptidases and shows that, in many cases, one aminopeptidase is enough to supply the needed functions. Moreover, the expression of all three of them appears to correlate with the genetic complexity with the exception of rodents where ERAP2 has been lost.

## Do the Three Aminopeptidases Have an Extracellular Role?

ERAP1 has been found to be secreted by activated macrophages and, in turn, to potentiate their activation (52). More recently, it has been shown that ERAP2 also can be secreted from human monocyte-derived macrophages (MDMs) in response to IFNγ/LPS stimulation and this corresponds to an increased CD8^+^ T cells activity (53). The mechanisms underlying these effects are not clear. However, the secretion of these molecules apparently occurs in activated macrophages and might have different outcomes: i.e., the triggering of a vascular response relevant in the inflammatory process accompanying these diseases. In humans for example, ERAP1 rs30187, a loss-of function gene variant that reduces AngII degradation *in vitro*, is associated with hypertension (54). The role of LNPEP in the RAS is well-documented as well as the association of ERAP2 with preeclampsia. We can therefore speculate that all three aminopeptidases play a role in the control of the vascular response in inflammation. In support of this theory there is their expression along the zoological scale where, in most cases, the “classical” antigen presenting functions are absent. It might be that the specific role in the complex network of antigen presentation has been acquired later on and maintained because the “trimming” function has become an essential part of a productive defense against viruses (18). A second implication is that the redundancy of their function can lead to a “quantitative” control of the three aminopeptidases so that the full expression of all of them is disfavored. In this regard, we have recently shown that the transcription of ERAP1 and ERAP2 is interlinked: the variant G at rs75862629 in the intergenic region between the two genes strongly influences the expression of the two aminopeptidases with a down-modulation of ERAP2 coupled with a significant higher expression of ERAP1 (40). Even more intriguing is the observation that the ERAP2 variants co-segregating with a lower or null expression of ERAP2 (G at rs75862629 and G at rs2248374, respectively) appear more frequent in the equatorial regions, where the malaria has been endemic. It is interesting that an evolutionary analysis of antigen presentation pathways (55) showed that positive selection has driven the recurrent appearance of ERAP2 protein-destabilizing variants during mammalian evolution in a region apparently not involved in antigen presentation. LNPEP was also found to evolve adaptively in mammals. In this case, four positively selected sites were found to be located at the C-terminal domain 4, which has been shown to possess regulatory activity. The same study points out an inter-species selection of LNPEP gene, an inter-and intra-species selection of ERAP2 gene and an intra-species selection only in the human lineage of ERAP1 gene. This suggests that the ERAP1 gene appeared consequently to that of ERAP2.

In conclusion, we propose here that the expression of the three aminopeptidases mapping on chromosome 5 in the human genome have evolved from the duplication of the precursor LNPEP gene. We also propose that their original function is in the control of the renin–angiotensin system and blood pressure. The “trimming” function has been acquired later on along the zoological scale. The most ancient function is therefore quantitatively controlled through an alternative expression in most cases. Indeed in mammals, ERAP2 has been sacrificed being absent in rodents and controlled in humans where 25% of population is null and 50% is monoallelic. These observations offer a new functional frame in which the association of the aminopeptidases with immune mediated diseases can be reconsidered.

## Author Contributions

FP has produced the data and the figures about the amino peptidases expression. FP, MF, and RS have written the review. This hypothesis originates from scientific discussions to which all authors have contributed. All authors contributed to the article and approved the submitted version.

## Conflict of Interest

The authors declare that the research was conducted in the absence of any commercial or financial relationships that could be construed as a potential conflict of interest.

## Supplementary Material

Supplementary Table 1 Species of mammals, birds, reptiles, amphibians, and bone fish included in the analysis for the presence or absence of the genes ERAP1, ERAP2, and LNPEP.
