# Supplementary material for: The Multifaceted Nature of Aminopeptidases ERAP1, ERAP2, and LNPEP: From Evolution to Disease
Source: Front Immunol. 2020 Jul 23;11:1576. doi: 10.3389/fimmu.2020.01576 (PMC7390905; doi:10.3389/fimmu.2020.01576)
Supplement: Supplementary Table 1 — Species of mammals, birds, reptiles, amphibians, and bone fish included in the analysis for the presence or absence of the genes ERAP1, ERAP2, and LNPEP. [file Table_1.DOCX]

| **MAMMALS** | ERAP1 | ERAP2 | LNPEP | **BIRDS** | ERAP1 | ERAP2 | LNPEP | **BONE FISHES** | ERAP1 | ERAP2 | LNPEP |
| --- | --- | --- | --- | --- | --- | --- | --- | --- | --- | --- | --- |
| *Acinonyx jubatus* (cheetah) | yes | yes | yes | *Struthio camelus australis* | yes | yes | yes | *Stegastes partitus* (bicolor damselfish) | yes | yes | yes |
| *Ailuropoda melanoleuca* (giant panda) | yes | yes | yes | *Apteryx row* (Okarito brown kiwi) | yes | yes | yes | *Gouania willdenowi* (blunt snouted clingfish) | no | yes | no |
| *Aotus nancymaae* (Mas night monkey) | yes | yes | yes | *Gallus Gallus* (chicken) | yes | no | yes | *Hippocampus comes* (tiger tail seahorse) | no | yes | no |
| *Balaenoptera acutorostrata scammoni* | yes | yes | yes | *Tinamus guttatus* (white-throated tinamou) | yes | no | yes | *Cyprinus carpio* (common carp) | no | yes | no |
| *Bison bison bison* | yes | yes | yes | *Nothoprocta perdicaria* | yes | no | yes | *Onchorhynctus mykiss* (rainbow trout) | no | yes | no |
| *Bos indicus* (zebu cattle) | yes | yes | yes | *Anser cygnoides domesticus* | yes | no | yes | *Onchorhynctus nerca* (sockeie salmon) | no | yes | no |
| *Bos indicus x Bos taurus* (hybrid cattle) | yes | yes | yes | *Anas platyrhynchos* (mallard) | yes | no | yes | *Boleophtalmus pectinirostris* (great blu-spotted *mudskipper*) | no | yes | yes |
| *Bos mutus* (wild yak) | yes | yes | yes | *Coturnix japonica* (Japanese quail) | yes | no | yes | *Anabas testudineus* (climbing perch) | no | yes | yes |
| *Bos taurus* (cattle) | yes | yes | yes | *Numida meleagris* (helmeted guineafowl) | yes | no | yes | *Monopterus albus* (swamp eel) | no | yes | yes |
| *Bubalus bubalis* (water buffalo) | yes | yes | yes | *Cariama cristata* (Red-legged seriema) | yes | no | yes | *Mastacembelus armatus* (zig-zag eel) | no | yes | yes |
| *Callithrix jacchus* (white-tufted-ear marmoset) | yes | yes | yes | *Aquila chrysaetos canadensis* | yes | no | yes | *Echeneis neucrates* (live sharksucker) | no | yes | yes |
| *Callorhinus ursinus* (northern fur seal) | yes | yes | yes | *Haliaeetus leucocephalus* (bald eagle) | yes | no | yes | *Parambassis ranga* (Indian glassy fish) | no | yes | yes |
| *Camelus bactrianus* (Bactrian camel) | yes | yes | yes | *Opisthocomus hoazin* | yes | no | yes | *Acanthochromis polyachantus* (spiny chromis) | no | yes | yes |
| *Camelus dromedarius* (Arabian camel) | yes | yes | yes | *Colius striatusspeckled* (mousebird) | yes | no | yes | *Larimichthys crocea* (large yellow croaker) | no | yes | yes |
| *Camelus ferus* (Wild Bactrian camel) | yes | yes | yes | *Charadrius vociferus* (killdeer) | yes | no | yes | *Takifugu rubripes* (torafugu) | no | yes | yes |
| *Canis lupus dingo* (dingo) | yes | yes | yes | *Cuculus canorus* (common cuckoo) | yes | no | yes | *Cottoperca gobio* | no | yes | yes |
| *Canis lupus familiaris* (dog) | yes | yes | yes | *Mesitornis unicolor* (brown roatelo) | yes | no | yes | *Pygocentrus nattereri* (red-bellied piranha) | no | yes | yes |
| *Capra hircus* (goat) | yes | yes | yes | *Nipponia nippon* (crested ibis) | yes | no | yes | *Tachysurus fulvidraco* (yellow catfish) | no | yes | yes |
| *Carlito syrichta* (Philippine tarsier) | yes | yes | yes | *Egretta garzetta* (little egret) | yes | no | yes | *Ictalurus punctatus* (channel catfish) | no | yes | yes |
| *Cebus capucinus imitator* | yes | yes | yes | *Pygoscelis adeliae* (Adelie penguin) | yes | no | yes | *Pangasianodon hypophthalmus*(striped catfish) | no | yes | yes |
| *Ceratotherium simum simum* (southern white rhinoceros) | yes | yes | yes | *Dromaius novaehollandiae* (emu) | yes | no | yes | *Clupea harengus* (Atlantic herring) | no | yes | yes |
| *Cercocebus atys* (sooty mangabey) | yes | yes | yes | *Athene cunicularia* (burrowing owl) | no | no | yes | *Denticeps clupeoides* (denticle herring) | no | yes | yes |
| *Chlorocebus sabaeus* (green monkey) | yes | yes | yes | *Calidris pugnax* (ruff) | no | no | yes | *Scleropages formosus* (Asian bonytongue) | no | yes | yes |
| *Colobus angolensis palliatus* | yes | yes | yes | *Pterocles gutturalis* (yellow-throated sandgrouse) | no | no | yes | *Paramormyrops kingsleyae* | no | yes | yes |
| *Condylura cristata* (star-nosed mole) | yes | yes | yes | *Columba livia* (rock pigeon) | no | no | yes | *Astatotilapia calliptera* (eastern happy) | no | yes | yes |
| *Dasypus novemcinctus* (nine-banded) | yes | yes | yes | *Leptosomus discolor* (cuckoo roller) | no | no | yes | *Electrophorus electricus* (electric eel) | no | yes | yes |
| *Delphinapterus leucas* (beluga whale) | yes | yes | yes | *Falco cherrug* (Saker falcon) | no | no | yes | *Nothobranchius furzeri* (torquoise killfish) | no | no | yes |
| *Desmodus rotundus* (common vampire bat) | yes | yes | yes | *Falco peregrinus* (peregrine falcon) | no | no | yes | *Lates calcarifer* (barramundi perch) | no | no | yes |
| *Elephantulus edwardii* (Cape elephant shrew) | yes | yes | yes | *Chlamydotis macqueenii* (Macqueens bustard) | no | no | yes | *Seriola dumerili* (greater amberjack) | no | no | yes |
| *Enhydra lutris kenyoni* | yes | yes | yes | *Lonchura striata domestica* (Bengalese finch) | no | no | yes | *Oncorhynchus kisutch* (coho salmon) | no | no | yes |
| *Eptesicus fuscus* (big brown bat) | yes | yes | yes | *Serinus canaria* (Common canary) | no | no | yes | *Poecilia latipinna* (sailfin molly) | no | no | yes |
| *Equus asinus* (ass) | yes | yes | yes | *Corvus cornix cornix* | no | no | yes | *Betta splendens* (Siamese fighting fish) | no | no | yes |
| *Equus caballus* (horse) | yes | yes | yes | *Corvus brachyrhynchos* (American crow) | no | no | yes | *Seriola lalandi dorsalis* | no | no | yes |
| *Equus przewalskii* (Przewalskis horse) | yes | yes | yes | *Neopelma chrysocephalum* (saffron-crested tyrant-manakin) | no | no | yes | *Paralichthys olivaceus* (Japanese flounder) | no | no | yes |
| *Erinaceus europaeus* (western European hedgehog) | yes | yes | yes | *Manacus vitellinus* (golden-collared manakin) | no | no | yes | *Amphiprion ocellaris* (clown anemonefish) | no | no | yes |
| *Eumetopias jubatus* (Steller sea lion) | yes | yes | yes | *Lepidothrix coronata* (blue-crowned manakin) | no | no | yes | *Labrus bergylta* (ballan wrasse) | no | no | yes |
| *Galeopterus variegatus* (Sunda flying lemur) | yes | yes | yes | *Corapipo altera* (White-ruffed manakin) | no | no | yes | *Perca flavescens* (yellow perch) | no | no | yes |
| *Gorilla gorilla* (western gorilla) | yes | yes | yes | *Pipra filicauda* (Wire-tailed manakin) | no | no | yes | *Salmo salar* (Atlantic salmon) | no | no | yes |
| *Hipposideros armiger* (great roundleaf bat) | yes | yes | yes | *Zonotrichia albicollis* (white-throated sparrow) | no | no | yes | *Erpetoichthys calabaricus*(reedfish) | no | no | yes |
| *Homo sapiens* | yes | yes | yes | *Empidonax traillii* (willow flycatcher) | no | no | yes | *Salmo trutta* (river trout) | no | no | yes |
| *Ictidomys tridecemlineatus* (thirteen-lined ground squirrel) | yes | yes | yes | *Ficedula albicollis* (collared flycatcher) | no | no | yes |  |  |  |  |
| *Jaculus jaculus* (lesser Egyptian jerboa) | yes | yes | yes | *Acanthisitta chlorisrifleman* | no | no | yes |  |  |  |  |
| *Lagenorhynchus obliquidens* (Pacific white-sided dolphin) | yes | yes | yes | *Geospiza fortis* (medium ground-finch) | no | no | yes |  |  |  |  |
| *Lipotes vexillifer* (Yangtze River dolphin) | yes | yes | yes | *Pseudopodoces humilis* (Tibetan ground-tit) | no | no | yes |  |  |  |  |
| *Loxodonta africana* (African savanna elephant) | yes | yes | yes | *Parus major* (Great Tit) | no | no | yes |  |  |  |  |
| *Macaca fascicularis* (crab-eating macaque) | yes | yes | yes | *Sturnus vulgaris* (Common starling) | no | no | yes |  |  |  |  |
| *Macaca mulatta* (Rhesus monkey) | yes | yes | yes | *Nestor notabilis* (Kea) | no | no | yes |  |  |  |  |
| *Macaca nemestrina* (pig-tailed macaque) | yes | yes | yes | *Aptenodytes forsteri* (emperor penguin) | no | no | yes |  |  |  |  |
| *Mandrillus leucophaeus* (drill) | yes | yes | yes | *Melopsittacus undulatus* (budgerigar) | no | no | yes |  |  |  |  |
| *Manis javanica* (Malayan pangolin) | yes | yes | yes | *Antrostomus carolinensis* (chuck-wills-widow) | no | no | yes |  |  |  |  |
| *Marmota flaviventris* (yellow-bellied marmot) | yes | yes | yes | *Chaetura pelagica* (chimney swift) | yes | no | no |  |  |  |  |
| *Marmota marmota marmota* (Alpine marmot) | yes | yes | yes | *Meleagris gallopavo* (turkey) | yes | no | no |  |  |  |  |
| *Microcebus murinus* (gray mouse lemur) | yes | yes | yes | *Haliaeetus albicilla* (white-tailed eagle) | yes | no | no |  |  |  |  |
| *Miniopterus natalensis* | yes | yes | yes | *Gavia stellata* (red-throated loon) | yes | no | no |  |  |  |  |
| *Monodon monoceros* (narwhal) | yes | yes | yes | *Fulmarus glacialis* (Northern fulmar) | yes | no | no |  |  |  |  |
| *Mustela putorius furo domestic ferret* | yes | yes | yes | *Buceros rhinoceros silvestris* | yes | no | no |  |  |  |  |
| *Myotis brandtii* (Brandts bat) | yes | yes | yes | *Balearica regulorum gibbericeps* (East African grey crowned-crane) | yes | no | no |  |  |  |  |
| *Myotis davidii* | yes | yes | yes | *Pelecanus crispus* (Dalmatian pelican) | yes | no | no |  |  |  |  |
| *Myotis lucifugus* (little brown bat) | yes | yes | yes | *Phaethon lepturus* (White-tailed tropicbird) | yes | no | no |  |  |  |  |
| *Neomonachus schauinslandi* (Hawaiian monk seal) | yes | yes | yes | *Picoides pubescens* (Downy woodpecker) | yes | no | np |  |  |  |  |
| *Neophocaena asiaeorientalis asiaeorientalis* (Yangtze finless porpoise) | yes | yes | yes | *Calypte anna* (Annas hummingbird) | yes | no | no |  |  |  |  |
| *Nomascus leucogenys* (northern white-cheeked gibbon) | yes | yes | yes | **LIZARDS** |  |  |  |  |  |  |  |
| *Odobenus rosmarus divergens* (Pacific walrus) | yes | yes | yes | *Pogona vitticeps* | yes | no | yes |  |  |  |  |
| *Odocoileus virginianus texanus* | yes | yes | yes | *Podarcis muralis* | yes | no | yes |  |  |  |  |
| *Orcinus orca* (killer whale) | yes | yes | yes | *Anolis carolinensis* | yes | no | yes |  |  |  |  |
| *Orycteropus afer afer* | yes | yes | yes | *Gekko japonicus* | yes | no | yes |  |  |  |  |
| *Ovis aries* (sheep) | yes | yes | yes | *Thamnophis sirtalis* | yes | no | yes |  |  |  |  |
| *Pan paniscus* (pygmy chimpanzee) | yes | yes | yes | *Notechis scutatus* | yes | no | yes |  |  |  |  |
| *Pan troglodyte* (*s*chimpanzee) | yes | yes | yes | *Pseudonaja textilis* | yes | no | yes |  |  |  |  |
| *Panthera pardus* (leopard) | yes | yes | yes | *Protobothrops mucrosquamatus* | yes | no | yes |  |  |  |  |
| *Panthera tigris altaica* Amur (tiger) | yes | yes | yes | *Python bivittatus* | yes | no | yes |  |  |  |  |
| *Papio anubis* (olive baboon) | yes | yes | yes | **TURTLES** |  |  |  |  |  |  |  |
| *Phyllostomus discolor* (pale spear-nosed bat) | yes | yes | yes | *Chelonia mydas* (green sea turtle) | | | |  |  |  |  |
| *Physeter catodon* (sperm whale) | yes | yes | yes | *Terrapene carolina triunguis* (three toed box turtle) | yes | no | yes |  |  |  |  |
| *Piliocolobus tephrosceles* (Ugandan red Colobus) | yes | yes | yes | *Chrysemys picta* (painted turtle) | yes | no | yes |  |  |  |  |
| *Pongo abelii* (Sumatran orangutan) | yes | yes | yes | *Pelodiscus sinensis* (chinese soft-shelled turtle) | yes | no | no |  |  |  |  |
| *Propithecus coquereli* (Coquerels sifaka) | yes | yes | yes | **ALLIGATORS** |  |  |  |  |  |  |  |
| *Pteropus alecto* (black flying fox) | yes | yes | yes | *Alligator mississippiensis* | yes | no | yes |  |  |  |  |
| *Puma concolor* (puma) | yes | yes | yes | *Crocodylus porosus* | yes | no | yes |  |  |  |  |
| *Rattus norvegicus* (Norway rat) | yes | yes | yes | *Alligator sinensis* | no | yes | yes |  |  |  |  |
| *Rhinopithecus bieti* (black snub-nosed monkey) | yes | yes | yes | *Gavialis gangeticus* | no | yes | yes |  |  |  |  |
| *Rhinopithecus roxellana* (golden snub-nosed monkey) | yes | yes | yes | **AMPHIBIA** |  |  |  |  |  |  |  |
| *Rousettus aegyptiacus* (Egyptian rousette) | yes | yes | yes | *Rhinatrema bivittatum* | yes | yes | yes |  |  |  |  |
| *Saimiri boliviensis* (Bolivian squirrel monkey) | yes | yes | yes | *Xenopus tropicalis* (tropical clawed frog) | yes | no | yes |  |  |  |  |
| *Sus scrofa* (pig) | yes | yes | yes | Nanorana parkeri | yes | no | yes |  |  |  |  |
| *Theropithecus gelada* (gelada) | yes | yes | yes |  |  |  |  |  |  |  |  |
| *Trichechus manatus latirostris* (Florida manatee) | yes | yes | yes |  |  |  |  |  |  |  |  |
| *Tupaia chinensis* (Chinese tree shrew) | yes | yes | yes |  |  |  |  |  |  |  |  |
| *Tursiops truncatus* (common bottlenose dolphin) | yes | yes | yes |  |  |  |  |  |  |  |  |
| *Urocitellus parryii* (Arctic ground squirrel) | yes | yes | yes |  |  |  |  |  |  |  |  |
| *Ursus arctos horribilis* | yes | yes | yes |  |  |  |  |  |  |  |  |
| *Ursus maritimus* (polar bear) | yes | yes | yes |  |  |  |  |  |  |  |  |
| *Vicugna pacos* (alpaca) | yes | yes | yes |  |  |  |  |  |  |  |  |
| *Vulpes vulpes* (red fox) | yes | yes | yes |  |  |  |  |  |  |  |  |
| *Zalophus californianus* (California sea lion) | yes | yes | yes |  |  |  |  |  |  |  |  |
| *Castor canadensis* (American beaver) | yes | no | yes |  |  |  |  |  |  |  |  |
| *Cavia porcellus* (domestic guinea pig) | yes | no | yes |  |  |  |  |  |  |  |  |
| *Chinchilla lanigera* (long-tailed chinchilla) | yes | no | yes |  |  |  |  |  |  |  |  |
| *Chrysochloris asiatica* (Cape golden mole) | yes | no | yes |  |  |  |  |  |  |  |  |
| *Cricetulus griseus* (Chinese hamster) | yes | no | yes |  |  |  |  |  |  |  |  |
| *Echinops telfairi* (small Madagascar hedgehog) | yes | no | yes |  |  |  |  |  |  |  |  |
| *Felis catus* (domestic cat) | yes | no | yes |  |  |  |  |  |  |  |  |
| *Fukomys damarensis* (Damara mole-rat) | yes | no | yes |  |  |  |  |  |  |  |  |
| *Heterocephalus glaber* (naked mole-rat) | yes | no | yes |  |  |  |  |  |  |  |  |
| *Mesocricetus auratus* (golden hamster) | yes | no | yes |  |  |  |  |  |  |  |  |
| *Microtus ochrogaster* (prairie vole) | yes | no | yes |  |  |  |  |  |  |  |  |
| *Monodelphis domestica* (gray short-tailed opossum) | yes | no | yes |  |  |  |  |  |  |  |  |
| *Mus caroli* (Ryukyu mouse) | yes | no | yes |  |  |  |  |  |  |  |  |
| *Mus musculus* (house mouse) | yes | no | yes |  |  |  |  |  |  |  |  |
| *Mus pahari* (shrew mouse) | yes | no | yes |  |  |  |  |  |  |  |  |
| *Nannospalax galili* (Upper Galilee mountains blind mole rat) | yes | no | yes |  |  |  |  |  |  |  |  |
| *Octodon degus* (degu) | yes | no | yes |  |  |  |  |  |  |  |  |
| *Oryctolagus cuniculus* (rabbit) | yes | no | yes |  |  |  |  |  |  |  |  |
| *Otolemur garnettii* (small-eared galago) | yes | no | yes |  |  |  |  |  |  |  |  |
| *Peromyscus leucopus* (white-footed mouse) | yes | no | yes |  |  |  |  |  |  |  |  |
| *Peromyscus maniculatus bairdii* (prairie deer mouse) | yes | no | yes |  |  |  |  |  |  |  |  |
| *Phascolarctos cinereus*koala | yes | no | yes |  |  |  |  |  |  |  |  |
| *Pteropus vampyrus* (large flying fox) | yes | no | yes |  |  |  |  |  |  |  |  |
| *Sarcophilus harrisii* (Tasmanian devil) | yes | no | yes |  |  |  |  |  |  |  |  |
| *Sorex araneus* (European shrew) | yes | no | yes |  |  |  |  |  |  |  |  |
| *Suricata suricatta* (meerkat) | yes | no | yes |  |  |  |  |  |  |  |  |
| *Vombatus ursinus* (common wombat) | yes | no | yes |  |  |  |  |  |  |  |  |
| *Dipodomys ordii* (Ords kangaroo rat) | no | yes | yes |  |  |  |  |  |  |  |  |
| *Meriones unguiculatus* (Mongolian gerbil) | no | yes | yes |  |  |  |  |  |  |  |  |
| *Ochotona princeps* (American pika) | no | yes | yes |  |  |  |  |  |  |  |  |
| *Ornithorhynchus anatinus* (platypus) | no | yes | yes |  |  |  |  |  |  |  |  |
| *Pantholops hodgsonii* (chiru) | no | yes | yes |  |  |  |  |  |  |  |  |
